# Medical abortion in Nepal: a qualitative study on women’s experiences at safe abortion services and pharmacies

**DOI:** 10.1186/s12978-019-0755-0

**Published:** 2019-07-15

**Authors:** Claire Rogers, Sabitri Sapkota, Rasmita Paudel, Jaya A. R. Dantas

**Affiliations:** 1 International Health Programme, Faculty of Health Sciences, Curtin University, Perth, 6102 Western Australia; 2 Marie Stopes International, London, UK; 3 Independent Health Research Consultant, Kathmandu, Nepal

**Keywords:** Safe abortion, Medical abortion, Post-abortion care, Contraception, Pharmacy, Nepal, SRHR

## Abstract

**Background:**

Although Nepal legalised abortion in 2002, a significant number of women continue to access unsafe abortions. An estimated 60% of all abortions performed in 2014 were unsafe, with unsafe abortion continuing to be a leading contributor to maternal mortality. Despite medical abortion access being solely permitted through government accredited safe abortion services, medical abortion pills are readily available for illegal purchase at pharmacies throughout the country.

**Methods:**

Utilising an Assets Focused Rapid Participatory Appraisal (AFRPA) research methodology, underpinned by a health information pyramid conceptual framework, this qualitative exploratory study collected data from in-depth, open-ended interviews. The study explored the medical abortion and sexual and reproductive health experiences of ten women who accessed medical abortion through an accredited safe abortion service, and ten women who accessed unsafe medical abortion through pharmacies.

**Results:**

Thematic content analysis revealed emerging themes relating to decision-making processes in accessing safe or unsafe medical abortion; knowledge of safe abortion services; and SRH information access and post-abortion contraceptive counselling. Findings emphasised the interconnectivity of sexual and reproductive health and rights; reproductive coercion; education; poverty; spousal separation; and women’s personal, social and economic empowerment.

**Conclusions:**

While barriers to safe abortion services persist, so will the continued demand for medical abortion provision through pharmacies. Innovated and effective harm reduction implementations combined with access and information expansion strategies offer the potential to increase access to safe medical abortion while decreasing adverse health outcomes for women.

**Electronic supplementary material:**

The online version of this article (10.1186/s12978-019-0755-0) contains supplementary material, which is available to authorized users.

## Plain English Summary

Although abortion is legal in Nepal, unsafe abortion is one of the leading causes of maternal death. Abortions are only allowed to be provided by trained health professionals at government approved services. Despite this restriction, medical abortion pills can be easily purchased at pharmacies throughout the country.

To understand the experiences of women who have had a medical abortion, 20 women were interviewed: ten who went to a safe abortion clinic and ten women who purchased medical abortion pills from a pharmacy. The interviews showed themes relating to why women go to safe or unsafe places to get a medical abortion; how women learned where to get the medical abortion pills from; what health information women were given; and if they were offered contraception at the time. The findings highlighted that many factors impact a woman’s decision to have a medical abortion, where she will get it from and if she will use contraception.

While women continue to face barriers to safe abortion services, there will be a demand for pharmacies to illegally sell medical abortion pills. Strategies are vitally needed to reduce the harm women face by purchasing medical abortion through pharmacies, as well as expanding ways information about safe medical abortion can be provided.

## Background

The World Health Organization (WHO) and Guttmacher Institute estimate that between 2010 and 2014, 56 million induced abortions occurred each year worldwide [1]. Of these abortions, 25 million unsafe abortions (45% of all abortions) occurred globally every year, with the majority of unsafe abortions (97%), occurring in developing countries in Africa, Asia and Latin America [2]. Abortion is considered *safe* when it is performed in accordance with WHO guidelines and standards, performed by a trained health worker using WHO-recommended methods appropriate to the pregnancy duration [2–5].

Reconceptualization of the framework and methodology for estimating unsafe abortion has further divided the WHO classification of *unsafe* abortion into two categories of *less safe* and *least safe* [5]. In their 2017 study, Ganatra et al. classified abortions as less safe if only one of two criteria were met: (1) the abortion was performed by a trained provider, however an outdated or unsafe method (e.g., sharp curettage) was utilised or (2) a safe method of abortion (e.g., mifepristone and/or misoprostol) was used, but was administered without adequate information or support from a trained provider. Least safe abortions are classified as abortions provided by untrained individuals using dangerous methods such as ingestion of caustic substances, insertion of foreign objects, and the use of traditional herbal mixtures or tonics [5]. Of the 25 million unsafe abortions (45% of all abortions) that occurred annually between 2010 and 2014, an estimated 17 million (31%) were considered less safe, and 8 million (14%) were least safe [2, 5].

For over four decades, political and social advocates from Nepal’s medical and public health communities, supported by women’s rights activists, pushed for the liberalisation of Nepal’s restrictive abortion laws, finally resulting in the legalisation of surgical abortion (manual vacuum aspiration) in 2002 and the legalisation of medical abortion (mifepristone and misoprostol) in 2009 [6]. Under the current law, abortion is permitted up to 12 weeks of gestational age on the request of the pregnant women, up to 18 weeks of gestational age in the case of rape or incest and at any gestational age if the pregnancy is detrimental to the women’s health and life or if there is foetal impairment [6, 7].

In Nepal, medical abortion (MA) is the most frequently accessed method of pregnancy termination (79%), followed by manual vacuum aspiration (17%) and dilation and evacuation/dilation and curettage (7%) [8]. However, legalisation of abortion alone has not been adequate to facilitate access to safe abortion services for all women, and many barriers to services remain [9–13]. Of the estimated 323,100 abortions performed in Nepal during 2014, nearly 60% (186, 100) were considered unsafe, having been carried out by untrained or unregistered providers or self-induced [14].

Faced with a number of social and cultural factors such as a patriarchal society, limited sexual reproductive health and rights (SRHR) autonomy and knowledge, geographic isolation as well as abortion stigma, many Nepali women remain unaware of the legal status of abortion and have limited or no knowledge of where to access safe abortion services [12, 15, 16]. In August 2016, the Government of Nepal announced a strategy to provide free safe abortion services in Government clinics, in combination with the provision of free family planning services, to help mitigate financial barriers to accessing safe abortion services [17–19]. However, without simultaneously implementing consistent and comprehensive monitoring and evaluation of services, it remains unclear if this strategy can genuinely provide high quality, effective and equitable safe abortion services to the women of Nepal [11].

The Government of Nepal registered MA brands (combined regime of mifepristone and misoprostol), have been available only on prescription through Government accredited safe abortion providers since 2009 [19, 20]. Despite these restrictions, registered and unregistered brands of MA are readily available for purchase at pharmacies, often referred to as chemists or medical shops [14]. The porous border between Nepal and India enables the illegal entry of unregistered MA brands as well as ayurvedic and traditional medicines with supposedly abortive properties, many of which are then sold illegally through pharmacies [20, 21]. Recent NHDS data shows that 19% of women who had an abortion reported receiving MA pills from a pharmacist [8]. While the Department of Drug Administration and collaborations between the Government, I/NGOs and the private sector have sought to reduce the illegal possession and sale of MA through pharmacies, it has proven challenging and to date, has been unsuccessful [11, 20]. Regardless of illegality, women in Nepal will continue to access MA through pharmacies, and while there is a high demand, pharmacies will continue to sell the pills [11]. Although it has been 15 years since the legalisation of abortion in Nepal, unsafe abortion remains the third highest (7%) direct cause of maternal death in Nepal [12].

Efficient and equitable provision of Post-Abortion Care (PAC) is an essential component for positive health outcomes for women who access safe abortion services and for the prevention of future unintended pregnancies [22–24]. However, in Nepal, women accessing MA through pharmacies do not systematically receive any form of PAC including adequate information regarding the administration of the drugs, SRH information, post-abortion family planning counselling or health care referral [11].

This qualitative, exploratory study aimed to provide a unique and in-depth analysis of the post-abortion experiences of women who have accessed MA through safe abortion services and women who have accessed unsafe MA by illegally obtaining the medication through pharmacies. This study explores post-abortion contraceptive counselling, access and use of contraception among Nepali women who purchased MA through pharmacies and offers a rich and detailed examination of their experiences and SRHR needs.

## Methods

Using an Assets Focused Rapid Participatory Appraisal (AFRPA) research methodology, underpinned by a health information pyramid conceptual framework, known as the Assets Focused Rapid Participatory Assessment Cycle (AFRPAC), this qualitative exploratory study utilised data collected from in-depth, open-ended interviews with 20 women from the Sunsari District of Nepal [25–27]. Participants included ten women who had accessed MA through a Marie Stopes Center (Clinic Clients), operated by Sunaulo Parivar Nepal, an implementing partner of Marie Stopes International Nepal (SPN/MSN) and ten women who had accessed medical abortion by illegally obtaining the tablets through pharmacies (Pharmacy Clients).

The methodology used in the study has an emphasis on assets and ensures that findings are concurrently solution- and problem-focused, as opposed to the identification of problems. Drawing on the perspectives of SRHR professionals and conversations with a cross-section of community members enabled the researchers to acquire a greater understanding of issues impacting women’s and girls’ SRHR within the local and national contexts and helped inform interview questions. [11, 28]. Complementing the qualitative findings, an analysis of current Government and non-Government SRH policy and clinical practices was concurrently undertaken.

### Participants

Inclusion criteria for participation in this study required women to be 15 to 49 years of age, able to speak and understand Nepali, able to give informed consent, live within the Sunsari District and have previously obtained MA pills for the termination of a pregnancy.

#### Clinic clients (CC)

Between September 2014 and April 2016 women attending the Itahari Marie Stopes Centre (MSC) for safe abortion services were informed of the study by clinic staff. Of those women, one hundred twelve shared their contact details and consented to be telephoned for potential research participation. Participants were purposively recruited from this sample of potential participants with 46 of these women meeting the inclusion criteria. To help mitigate recall bias, women who had accessed MA 3 to 6 months prior to data collection were selected. Contact was attempted with 23 women with a final ten women participating in the research. CC clients received the Nepal Government registered brand of MA - Mariprist (a mifepristone and misoprostol combi-pack).

#### Pharmacy clients (PC)

Between April and May, 2016, Female Community Health Volunteers (FCHVs) who were working closely with the MSC utilised their contacts within the study community to purposively recruit ten women who met the inclusion criteria. Due to difficulties with participant recruitment from this hard to reach community-based populations, MA access prior to data collection for PC ranged between approximately 3 weeks to 2 years. The medication PC participants obtained from a pharmacy for terminating their unwanted pregnancy is referred to as MA throughout this paper. However, it is impossible to ascertain whether they received a Nepal registered brand of MA, an unregistered, but legitimate brand of MA, counterfeit MA or a type of ayurvedic medicine with abortive properties in pill form [20].

### Data collection

The interviews were conducted between April and May, 2016, by the first and third authors, who are both female. A research information sheet, detailing the purpose and process of the study as well as an informed consent form was provided to all participants for review prior to interviews. Participants were encouraged to ask questions relating to their role in the study as well as any component of the research. After obtaining written or verbal informed consent, interviews of approximately 1 h in duration were conducted with the participants in a convenient, private location. Eighteen women provided written informed consent and two women, who were not literate, provided verbal informed consent. All interviews were conducted in Nepali as this was the preferred language of the participants. Throughout the interviews, the third author (fluent in both Nepali and English) translated interviewee responses in English to ensure both interviewers were able to ask questions and had a comprehensive understanding of the participants’ responses. Reflective field notes were taken throughout the interviews.

### Data analysis

Audio files were translated and transcribed after the interviews by the third author and a professional transcriptionist. A thematic analysis of in-depth interview content utilising NVivo Software was undertaken by the first author, with the first three authors reading the transcripts and discussing data saturation before collaboration and refinement of themes [29]. Thematic analysis enabled the examination of emergent themes within the data, facilitating the synthesis of overarching themes, themes and sub-themes [30, 31]. Analysis of interview content produced five overarching themes, 12 themes and 59 sub-themes as detailed in Additional file 1: Thematic Content Analysis of Interview Transcripts (*n* = 20).

Rigour in qualitative research is assessed within the context of dependability, credibility, confirmability and transferability, and trustworthiness [32–34]. To enhance the credibility and overall trustworthiness of this research, systematic checking, ongoing interpretation of data and an audit trail ensured information relating to the study design, methods and analysis were documented to allow for future replication [35–37]. Pilot testing of guiding questions was conducted with all authors assisting in the refinement and finalisation of the questions prior to the interviews.

### Ethical considerations

Ethical approval for this study was granted by the Nepal Health Research Council (NHRC 20/2014) as well as the Curtin University Human Research Ethics Committee (HR 17/2014), and informed consent was a prerequisite of research participation [38]. Ensuring cultural safety (aligned with beneficence), was of paramount importance, and it was essential that participants felt their voices were heard, that they were respected, and that they felt safe within the context of the research process [28, 38, 39].

Keeping with the ethical principal of non-maleficence, interviews with PC respondents did not focus on the legality of MA supply through pharmacies so as not to cause discomfort or distress to participants. As well as confirming respondents had a clear understanding of their role within the research and the purpose of the research, to ensure the ethical principles of justice and beneficence were adhered to, they were also informed of how their participation will assist the development of tangible SRHR policy and practice outcomes [28, 38]. To ensure the confidentiality of participants, generic titles detailing whether the participant was a CC or PC, a participant chosen pseudonym and a participant’s reported age, have been used throughout this article [38]. As many participants had to travel to contribute to the research, all respondents received 500 Nepali Rupees (NPR) of transport reimbursement as well as a light meal with refreshments before the interviews. To thank respondents for their time, all received a voucher for a free health check-up (detailed as a ‘general health check-up and valued at 150 NPR) and free pregnancy test (for privacy reasons, detailed as ‘women’s health check-up’ and valued at 100 NPR) at the NGO collaborating with the research. Initial findings of the research were disseminated at a community and stakeholder event in July 2016 in the Sunsari District.

## Results

Thematic content analysis of the in-depth interviews revealed emerging themes relating to safe abortion service provision, knowledge of safe abortion services, SRH information access, contraception counselling, geographical isolation, as well as socioeconomic and sociocultural factors. Research findings highlight the interconnectivity of SRHR; gender-based violence in the form of reproductive coercion and son preference; education; poverty; spousal separation; and women’s personal, social and economic empowerment. Table 1 details participant demographic information and Table 2 details participant reproductive health information.

**Table 1:** Participant Demographic Information

	Clinic Clients *(n = 10)*	Pharmacy Clients *(n = 10)*	ALL Participants *(n = 20)*
Age			
20–25 years	3	3	6
26–30 years	4	3	7
31–35 years	1	2	3
36–40 years	2	2	4
Married			
Yes	10	10	20
Husband Works Away^*^			
Yes	4	6	10
No	6	4	10
Family Structure^+^			
Single	6	6	12
Joint	4	4	8
Highest Level of Education			
No Primary	0	1	1
Some Primary	2	2	4
Some High School	3	5	8
Obtained SLC^^^	4	1	5
Obtained Bachelor’s Degree	1	0	1
Obtained Master’s Degree	0	1	1
Language/s Spoken at Home			
Nepali	7	9	16
Nepali and Maithili	2	1	3
Nepali and Hindi	1	0	1
Religion			
Hindu	10	8	18
Kirat Mundhum	0	1	1
Buddhist	0	1	1

* Husband works away from the family home either within Nepal or overseas

+ Single Family comprises of husband, wife and children. Joint Family comprises of husband, wife and children as well as members of the extended family (e.g. grandparents, uncles, aunties, nephews, nieces etc.) living in the one family home

^ School Leaving Certificate obtained on completion of high school

**Table 2:** Participant Reproductive Health Information

	Clinic Clients *(n = 10)*	Pharmacy Clients *(n = 10)*	ALL Participants *(n = 20)*
MA use prior to interview^#^			
< 3 months	0	2	2
3–6 months	10	2	12
6 months – 1 year	0	1	1
1–2 years	0	3	3
2–3 years	0	2	2
Incomplete abortion after MA use			
Yes	1	3	4
No	9	7	16
Contraceptive use directly prior to MA			
Yes	**3**	**1**	**4**
OCP^a^	1	0	1
Intermittent OCP	1	1	1
Intermittent Injectable^b^	1	0	1
No	**7**	**9**	**16**
No- wanted to conceive^*^	1	0	1
Contraceptive use directly after MA			
Yes	**10**	**2**	**12**
Condoms	1	0	1
OCP	5	0	5
Injectable (Depo^bb^)	0	1	1
Implant^c^ (Norplant^cc^)	4	0	4
IUD^d^ (Copper T^dd^)	0	1^+^	1
No	**0**	**8**	**8**
Current contraceptive use			
Yes	**8**	**3**	**11**
Condoms	1	0	1
Intermittent OCP	2	0	1
OCP	1	0	2
Depo	0	1	1
Norplant	4	1	5
Copper T	0	1	1
No	**2**	**7**	**9**
No - wants to conceive ^^^	0	1	1
Previous abortion/s			
None	8	9	17
One	1	1	2
Two	1	0	1
Previous miscarriage/s			
None	6	10	16
One	4	0	4
Number of deceased children			
None	10	8	18
One	0	1	1
Two	0	1	1
Number of living children			
One	4	2	6
Two	5	6	11
Three	0	1	1
Four	1	1	2
Child/children sex-mix			
Female and Male	3	5	8
Only Female	4	3	7
Only Male	3	2	5
Age when married			
Under 18 years	2	2	4
18–20 years	4	5	9
21–23 years	4	0	4
24–26 years	0	1	1
Not Recorded	0	2	2
Age at birth of first child			
Under 18 years	1	1	2
18–20 years	3	4	7
21–23 years	4	2	6
24–26 years	2	1	3
Not Recorded	0	2	2

# Approximate timeframe between the participant taking MA and their in-depth interview

a Oral Contraceptive Pill

b Hormonal contraceptive injection

bb Hormonal contraceptive injection brand

c Hormonal contraceptive implant

cc Hormonal contraceptive implant brand

d Intrauterine Device

dd Non-hormonal intrauterine device

* The total count for participants not using contraception when they fell pregnant before their subsequent MA includes one participant who was not using contraception as she wanted to become pregnant at that time

+ Contraceptive method was not obtained through a pharmacy. Method was inserted at an SRH Clinic shortly after taking MA

^ The total count for participants currently not using contraception includes one participant who is not currently using contraception as they want to become pregnant

### Abortion decision-making process

Both CC and PC respondents shared their reasons for obtaining an abortion and the driving forces behind their decision to terminate their pregnancies. Of the 20 women who accessed MA, 19 were due to unplanned pregnancies, and one (CC participant) required a termination as the foetus was no longer viable. Respondents highlighted the interconnectivity of sociocultural and socioeconomic factors that informed their decision-making process. Child spacing was a deciding factor for several participants living within both single and joint families. Participants emphasised the difficulties faced by women in their community to maintain expected gender roles within the home environment, while also striving for personal and economic empowerment.

*“I was pregnant too soon. My husband also did not want to have (another) baby immediately… We have many members in our family, women like us have to manage the house, so it was difficult.”* ***PC IDI 10, Uma, 25 years***

It is seen as a women’s duty to be the primary carer of children in Nepal and, although many joint households have multiple female family members to support, many other mothers undertake parenting duties alone. One participant shared her experience of being the sole carer of her children while pregnant and suffering from health concerns.

*“I had very young children. I had continuous vomiting. I had to be admitted to hospital, and it was difficult to take care of my children, so I aborted.”* ***PC IDI 8, Munna, 30 years***

Financial implications of an unwanted pregnancy were highlighted as key motivator in the abortion decision-making process for many of the respondents. One participant shared her experience of financial insecurity when her son was born and the impact this experience had on her decision to terminate her next pregnancy.

*“I have no money. I already had son. I had to take loans during his birth… We had to keep him in the intensive care unit. I had to invest a lot of money, I have taken loans, so I do not want to give birth again. If I give birth again, it will cost.”* ***CC IDI 8, Katrina, 20 years***

For participants from lower socioeconomic families, their unexpected pregnancy was a cause of financial anxiety. Being able to financially provide for children was a recurring theme throughout the interviews, with multiple respondents sharing their inability to care for and educate the children they have if they were to have another. Several participants expressed the necessity for their children to have access to schooling that they did not have, in order for their children to have a happy and successful life.

*“I did not want any more children, I have son and daughter… We are poor people, we should manage food for those children. Giving birth is not enough, we have to care for them, educate them.”* ***PC IDI 4, Parvati, 30 years***

Themes of reproductive coercion were frequently reiterated throughout interviews with both CC and PC participants. One respondent highlighted the sociocultural struggle many women in Nepal face when it comes to their reproductive autonomy and their ability to decide if, and when, they wish to fall pregnant.

*“My daughters and son are already grown up, but my husband still thinks I should (continue) to give birth... I thought that if it will be aborted with medicine, despite there will be bleeding, I’ll use that… I did not tell this to my husband.”* ***PC IDI 2, Reena, 40 years***

Within the patriarchal Nepali society, son-preference is common, and participants frequently commented on the pressure from husbands and mothers-in-law to conceive sons. Several respondents shared their happiness with only having daughters. While they did not personally feel they needed to have sons, one participant spoke of her husband’s concern of isolation they will experience in later life when their daughters get married and leave home.

*“My husband says: ‘don’t use (contraception) since we have only daughters’… Such desires will be in males only, he wants a son. For woman like me, it’s equal… Even if I don’t have desire (for more children), because of the force of husband, I need to give birth. Among four daughters two already got married. Another two also will go. After they go, only we two will be alone. Males think that way. I already have a granddaughter, I don’t want (more children).”* ***PC IDI 1, Sapana, 36 years***

While none of the respondents based their personal abortion decision-making process from having learnt the sex of the foetus through ultrasound technology, several participants shared stories relating to sex-selective abortions within their communities or extended families.

### Medical abortion access and uptake: safe abortion services vs pharmacies

#### Clinic clients

CC participants frequently stated that advice from friends and family members played a determining factor in their decision to access MA through a safe abortion provider.

*“My friend knew about the clinic (MSC). I shared my problem with her, so she suggested that I go there.”* ***CC IDI 10, Pratima, 25 years***

One CC participant shared that advice from a health care professional helped her decision making regarding where to access MA.

*“I initially thought about taking medicine from local medical store but there is a doctor living in our home, he explained to me that it is not safe to take medicine this way and suggested I go here (MSC).”* ***CC IDI 5, Geeta, 28 years***

Fear of negative health outcomes from accessing MA through a pharmacy played a key role in the decision-making process for several CC participants. This fear was often the result of learning of others’ negative experiences with pharmacy-supplied MA. Concerns of inadequate medical support and referral mechanisms (should they encounter complications) at pharmacies was reiterated by respondents as key factors in their decision to attend a safe abortion service.

*“First, I did not know about the clinic (MSC). My friends took medicine (MA) from a pharmacy. For one of my friends it worked, and for my other friend it did not. When it was not completely aborted, the same pharmacy where she purchased the medicine (MA) suggested her to go to (MSC)… It’s not good practice to go to the pharmacy for such medicine (MA)... After we give them money the pharmacy people will give us medicine. Whether it will be completely aborted or not will be at our own risk.”* ***CC IDI 10, Pratima, 25 years***

Concerns regarding the perceived dangers of inducing an abortion as well as the medical competency of those administering MA also played into the decision-making process for several participants to attend a safe abortion service for their abortion.

*“If the service is taken in a good place after consulting with an expert in this area, it is safer…We have heard many people saying somebody took medicine for abortion and is dead because of it or is having heavy bleeding now… Does that pharmacy person have enough idea about the medication to use, current month of pregnancy? No. So it is nowhere comparable with the consultation in hospitals or clinics with experts.”* ***CC IDI 4, Roghini, 32 years***

Previous experience of accessing MA through a pharmacy and having an incomplete abortion was also stated as being a driving force behind one CC participant attending the MSC for her second abortion.

*“(For my) first abortion some of my friends said that they used medicine from pharmacy for abortion, so they told me: ‘why you will go to hospital, go in the pharmacy and take it’… They (pharmacy) did not tell (me) anything about family planning. They just gave medicine for abortion… When I took medicine, it was not aborted completely, so I asked them ‘what to do?’ They suggested I go here (MSC).”* ***CC IDI 8, Katrina, 20 years***

The participant goes on to explain that her positive experience at the MSC for her previous incomplete abortion affected her decision making when she had a second unplanned pregnancy.

*“It is far (to travel) but here the service is good. They provide proper counselling which I cannot find in pharmacy. Whether here (MSC) or there (pharmacy), I have to pay, so I’ll come here.”* ***CC IDI 8, Katrina, 20 years***

Nine CC respondents attended the MSC to obtain their MA in the first instance, and one CC participant attended the MSC for MA due to an incomplete abortion after taking pharmacy provided MA.

#### Pharmacy clients

Similar to the CC participants, advice from people within the community regarding MA access was a considerable influence on PC respondents’ abortion seeking behaviour. One PC participant shared that a FCHV informed her she could purchase MA through a pharmacy. Another PC respondent was told of the ability to access MA through a pharmacy by a member of the pharmacy staff.

*“While buying the pregnancy test kit (at the pharmacy), I came to know (through information provided by a staff member) that I can buy that medicine (MA) there.”* ***PC IDI 8, Munna, 30 years***

Positive experiences of friends, family and neighbours in accessing MA through pharmacies was repeatedly stated as a deciding factor for PC participants choosing to purchase MA through a pharmacy.

*“I felt that will be easier. I had heard from others that they had taken (MA) and it worked well, so I thought it will be effective.”* ***PC IDI 8, Munna, 30 years***

Lack of nearby safe abortion services was highlighted by several PC participants as the reason they decided to access MA through a local pharmacy, with one participant highlighting the fear and worry associated with not being able to access SRH services.

*“I was alone that time, in a remote place. I did not have friends to take me to the hospital… I had a friend (working) in pharmacy so I got (MA) through them… If I have another unplanned pregnancy maybe I’ll go to a place with more facilities but if I am in a remote place, I will again have to go to a pharmacy.”* ***PC IDI 9, Bipana, 31 years***

Five of the ten PC participants did not physically purchase the MA from the pharmacy themselves. Instead, their husbands (three respondents), a female neighbour (one respondent) and a female friend who works at a pharmacy (one respondent) purchased the pills and relayed information to the women.

*“I did not know that we could buy those (MA) in government hospitals, I knew that it can be found in medicals (pharmacies)… I requested one sister (female friend) to bring… she works in that pharmacy.”* ***PC IDI 9, Bipana, 31 years***

### Medical abortion experience: safe abortion services vs pharmacies

CC and PC participants shared their experiences of obtaining MA and their thoughts and emotions towards the process. Universally, CC participants reported feeling supported and informed at the time of their clinic appointment and while several respondents spoke of feeling scared, said MSC clinic staff were able to reassure them. Fear of complications was also reported by PC participants who generally reported that their fear subsided only when the abortion was complete.

*“I was worried what problems might arise. I was also scared whether it will be completely aborted or not. I had heard of some women’s death due to complications.”* ***PC IDI 9, Bipana, 31 years***

In contrast, one PC participant shared how support from her husband and the pharmacy worker providing the MA (combined with information provided to her husband by the pharmacy worker), reassured her that her fear of post-abortion complications was unwarranted.

*“I was scared of taking that medicine (MA). He (husband) gave me consolation not to be scared (and told me the) pharmacy people said that it won’t be that bad.”* ***PC IDI 5, Sita, 25 years***

While all CC participants said they would access a safe abortion services again should they have a future unplanned pregnancy they wished to terminate, PC participants responses varied regarding future MA access. While most PC participants noted they would attend a safe abortion service, one participant shared that due to limited access to safe abortion services she would again access MA through a pharmacy, and one PC participant shared she would purchase MA through a pharmacy again due to her positive previous experience.

*If in case (of another unplanned pregnancy), maybe I’ll go to the same place (pharmacy) because it worked well for me.* ***PC IDI 8, Munna, 30 years***

Three PC participants had incomplete abortions after taking pharmacy provided MA requiring access to medical facilities. One PC participant shared her experience of taking multiple doses of MA before going to a health facility.

*It (MA) did not work. I thought it might take time. I told the person who brought it (female friend) and they said that it will be aborted after 2-3 days. But it was not aborted. So that person gave me another medicine. It was not aborted even with next dose of medicine… I thought it will be easily aborted but it did not happen.* ***PC IDI 4, Parvati, 30 years***

Several PC respondents highlighted the lack of medical referral information in the case of post-abortion complications. Concerns regarding the saftey and effectiveness of pharmacy provided MA were raised, as was the medical competency of those providing it.

*“They (pharmacy workers) should not do (administer MA) according to guess… There should be trained health worker working there. Some women’s life might be at risk… due to such medicines.”* ***PC IDI 2, Reena, 40 years***

### Post-abortion contraception and SRH information access and uptake: safe abortion services vs pharmacies

#### Clinic clients

All CC respondents reported receiving SRH information and contraceptive counselling during their appointment at the MSC (such as MA administration, what to expect from taking MA and possible side-effects, post-abortion follow up, contraceptive use education, and fertility desire discussions) with many displaying detailed recall of SRH information provided during their appointment. Several participants expressed the empowerment they now feel regarding their sexual and reproductive health.

*“(At my appointment) I became more knowledgeable about reproductive health. I am able to make self-decisions now. I can plan my family accordingly… They (MSC staff) gave detailed counselling… and information about contraceptives.”* ***CC IDI 5, Geeta, 28 years***

Several participants highlighted the impact post-abortion family planning counselling had on relieving their concerns around contraceptive use such as side-effects and fear of future unplanned pregnancies.

*“There were many things I did not know before which I learnt through this information… I don’t have to be scared (of unplanned pregnancy) while in sexual relation after the use of Norplant (Implant).”* ***CC IDI 1, Sabitri, 36 years***

Of the ten CC participants, four took up LARC (long-acting reversible contraception) and six accepted short-term modern contraceptive methods post-MA. Four had Implants inserted immediately after taking the first MA tablet, and all reported continuing the method at the time of their interview. One CC participant accessed condoms after their MA and reported continuing to use this method. Five CC participants accepted OCP at the time of their MA, with one reporting continuous current use, two intermittent use and two currently not using any form of contraception.

#### Pharmacy clients

Although women in the CC sample expressed relatively similar experiences relating to SRH information and contraceptive counselling provided by clinic staff during their appointments, the PC participants who purchased the MA themselves shared varied accounts of SRH information provision. Several respondents reported they received no SRH information other than when to take the MA tablets, which at the time, caused them concern.

*He asked me why I wanted to use medicine. I answered him the reason that my child was small, so he gave the medicine. He did not give me any other information… He just told me that it will work for some persons and for some it will not… I think that I should have gotten more information… Like the side effects of using that medicine.”* ***PC IDI 3, Sita, 28 years***

In contrast, other PC respondents reported being provided with SRH information at the time they purchased the MA such as MA administration, what to expect from taking MA and possible side-effects, and what to do if the abortion is incomplete (e.g., return to the pharmacy, go to a health clinic or hospital or call the pharmacy for support). However, only two participants discussed post-abortion contraceptive use with the pharmacy staff providing their MA.

*“They gave information on how to take the medicine… and side effects… They said that this medicine works for some people and for some people it won’t be completely aborted so in such case should go to hospital… Because of that (information) I have knowledge now. They suggested I use Depo (Injectable)… so I used Depo but it did not react well with me...now I use Norplant (Implant).”* ***PC IDI 6, Bharati, 25 years***

One PC client shared her experience of accessing MA through a pharmacy with her husband and highlighted the necessity for effective, culturally appropriate contraceptive counselling. The couple received information on MA administration, what to expect and what to do should complications arise as well as information about contraception. However, while the respondent felt the information on MA was useful, she expressed distress regarding the MA provider’s reaction to her not wanting to use post-abortion contraception.

*“They (pharmacy staff) informed us (about MA)… They told us (about contraception) and showed us the different methods. I already had used them and they (had) caused problems so we replied that we would discuss the matter… They yelled at us ‘why are you are not using contraceptives and are doing abortion?’ They (pharmacy staff) were angry with us for not using contraception.”* ***PC IDI 7, Kalpana, 35 years***

While other PC respondents stated they would have like to have received advice on post-abortion contraception at the time of their MA and indeed, post-abortion contraception information should have been provided to them, two PC participants stated they did not expect to receive this type of information from a pharmacy worker. Similar to the women who purchased the MA themselves, SRH information access varied in content for participants who did not purchase the MA. Several women reported only being told when to take the pills and to expect heavy bleeding, while other participants recollected more detailed SRH information provision and support from pharmacy workers.

Only one of the ten PC participants reported accessing a pharmacy provided method of contraception post-MA. This PC participant accessed pharmacy provided injectables, reporting they later changed to an Implant (inserted at a health clinic). One PC respondent had an intrauterine device (IUD) inserted at a health clinic shortly after her MA and was still using this contraceptive method. Eight PC respondents reported no contraception use post-MA.

Both CC and PC participants shared their post-abortion contraceptive decision-making process. Overwhelmingly, PC participants reported similar reasons for not using a modern method of contraception post-abortion, the recurring themes being previous negative contraception experiences and not having found a method that suited them.

*“(My) sister in law told me to use injection so I used injection. But I had excessive bleeding. I used pills, it also did not work. They inserted something (Implant) but I did not like it and I haven’t used anything after that.*” ***PC IDI 1, Sapana, 36 years***

PC respondents also reiterated fear of perceived side effects and lack of information about contraception as a reason for not accessing post-abortion contraception. In contrast to PC participants, CC respondents reported information and support they received through contraceptive counselling during their SRH clinic appointment, reassured them of their ability to choose a contraceptive method that would suit them.

*“They (MSC staff) counselled me about various methods like pills, Norplant (Implant), Copper-T (IUD) etc. I wanted to try pills first and if it doesn't suit me I can go for other alternatives as well… Now I know which of the family planning methods suit me. Otherwise I might not use contraceptives, I might again be pregnant, and have to go for another abortion.”* ***CC IDI 4, Roghini, 32 years***

Half of all respondents (CC = 4, PC = 6) shared that their husbands did not typically reside at the family home due to work commitments. Inconsistent contraceptive use, specifically OCP, or no contraceptive use, due to spousal separation was a recurring theme for several participants both before and after their unwanted pregnancies.

*“I got pregnant when I was still on pills (OCP). It's been around 5-6 years (I’ve been using pills). Whenever my husband is here I take pills and when he goes abroad I stop taking it… You know my husband lives around one month here and the next three months abroad, so I don't prefer Depo (Injectables) or Copper-T (IUD).”* ***CC IDI 1, Sabitri, 36 years***

At the time of their interviews, of the 10 participants whose husbands work away from the family home (either in another part of the country or overseas), four reported being on no form of contraception (CC = 1, PC = 3). Two respondents reported intermittent OCP use (CC = 2), three were using LARCs: two had an Implant inserted (CC = 1, PC = 1) and one participant had an IUD (PC = 1), and one participant was not using contraception as they wished to fall pregnant (PC = 1).

PC respondents reported overall limited contraceptive use at the time of data collection in comparison to their CC counterparts (CC = 8 and PC = 2). At the time of their interviews, one PC respondent was not using any contraceptive method as she wanted to conceive, and one respondent reported current use of injectables. Of the ten PC respondents, six reported currently not using a modern contraceptive method, although they did not currently wish to conceive.

### Contraception and SRH information access and uptake: general

The contrast in contraceptive and SRH information access between the CC and PC groups at the time of accessing MA highlights the difference in quality and consistency of service provision. Participants from both groups shared their experience and opinions relating to contraception and SRH information access and uptake outside of their immediate MA experience. Both CC and PC participants reported various reasons for not using contraception prior to their unwanted pregnancy, with the majority citing previous negative experiences with contraception use. Many participants also spoke of their continuing concerns based on the negative contraceptive experiences of other women.

*“I heard that pills will cause injuries in uterus. I heard that three months injection causes heavy bleeding. I heard that Norplant (Implant) will cause cancer, so I haven’t used any of these.”* ***PC IDI 8, Munna, 30 years***

Along with issues of reproductive coercion, the sociocultural pressure for Nepali women to have children was also frequently reported by both CC and PC respondents. Pressure from husbands relating to contraceptive use was also a reported factor for both CC and PC participants not using a modern method of contraception prior to their unplanned pregnancy and for several respondents, after their MA.

“*I told my husband that I’ll use the injection, but he did not allow… he said that we will be cautious, so I didn’t use (any contraception).”* ***PC IDI 5, Sita, 25 years***

Participants shared they will often base reproductive health decision, such as the type of contraceptive method they wish to use or where to access this method from, on information they receive from female family members and friends. Conversations with husbands and family (specifically mothers, mothers-in-law, sisters and sisters-in-law) as well as friends were reported as a means of acquiring information on SRH. Outside of an SRH clinic (government, I/NGO or private) or hospital environment, participants stated other ways they receive SRH information and SRH service provider information included TV, radio, facebook and advertising signs and billboards.

*“(I learnt about MSC services) from TV, radio and it is also written on signboards… Also, one of our friends keeps on searching and sharing… (SRH) information on Facebook which I feel very informative and useful.”* ***CC IDI 1, Sabitri, 36 years***

However, several participants highlighted the socioeconomic disparities in SRH and contraception information access, particularly for those living in rural and remote regions.

*“We don’t have TV or radio. (Female Community Health Volunteer) provides me information about contraceptives, but my husband does not want me to use.”* ***PC IDI 4, Parvati, 30 years***

Female Community Health Volunteers (FCHVs) were the most frequently cited source of SRH information and education as well as contraceptive access. Both CC and PC respondents emphasised the importance of FCHV within their communities, and how they feltsupported and comfortable with them.

*“I get information from health volunteers (FCHV). They are from our community, we will be close with them and can share.”* ***PC IDI 7, Kalpana, 35 years***

Participants also spoke of the critical role FCHVs play in providing SRH services to women living in rural and remote regions.

*“She (FCHV) works in a health post. She asks us if we have any problem and she travels around.”* ***PC IDI 1, Sapana, 36 years***

Many CC and PC participants expressed an overall lack of access to SRH information within their daily lives, with sociocultural issues including gender discrimination and SRH stigma stated as key inhibitors to SRH information access.

## Discussion

Women-centred safe abortion services support women to holistically exercise their SRHR by providing them with information, education and choice [40]. In contrast to unsafe services, they address both the physical and emotional reproductive needs of women, including contraceptive counselling, to support women in their MA journey [40]. Whether accessing MA through a safe abortion service or by unsafe means, reasons informing a woman’s decision to seek an abortion often highlight the intersectoral nature of SRHR, cultural and gender roles and socioeconomic status [8, 41–43].

Although the literature shows continuation of education as a prominent factor in abortion seeking decision making, particularly for younger women, our findings highlight the desire to ensure access to education for the children that women already have and is closely linked with concerns regarding the capacity to financially provide the same for another child [8, 42]. Findings from a 2017 study across 14 countries show that while women have abortions for a variety of reasons, the most frequently cited reasons for having an abortion are socioeconomic concerns or limiting childbearing [42].

Reproductive coercion is behaviour that inhibits a woman’s sexual and reproductive autonomy [44] and in Nepal, like many other developing countries in Asia, is often linked with son-preference [43–48]. Our findings highlight the complexity reproductive coercion has on women’s abortion seeking decision making. Research on sex-selective abortion in Nepal remains scarce. However, emerging evidence suggests such abortions are becoming increasingly common and therefore must be a consideration within all safe abortion and post-abortion family planning strategies [11, 47, 49, 50].

Effective culturally-safe contraceptive counselling is an essential component of PAC and assists women to space births, prevent future unwanted pregnancies and avoid unsafe abortion [22, 23]. It also plays an essential role in ensuring women’s concerns relating to contraceptive use are addressed, enabling women to make informed decisions regarding which method best suits them [51]. Informed choice increases the likelihood of post-abortion contraceptive use and empowerment to change methods, rather than total contraceptive discontinuation [24, 51–53].

With access to a wide range of contraceptive methods combined with comprehensive SRH information and education, contraception uptake in women post-abortion has shown to increase, however, many barriers remain [23]. Multiple studies conducted in Nepal on post-abortion contraception access and uptake through government, private and NGO SRH services highlight that even at registered safe abortion services, post-abortion contraception access and uptake remains a challenging aspect of effective PAC provision in Nepal [51, 52, 54, 55].

Effective and safe MA provision by non-physician clinicians is well documented [56–61]. Puri et al. (2018) and Rocca et al. (2018) demonstrate in their study on auxiliary nurse-midwife provided MA through pharmacies, that when pharmacy provided MA is administered by qualified health professionals effective and safe MA provision can be accomplished without compromising contraceptive care [53]. With many mid-level health providers (nurses and auxiliary nurse-midwives) proprietors of pharmacies in Nepal, their study highlights another promising avenue for safe and convenient MA provision and expansion through pharmacies [53, 61].

Between 2000 and 2014, trends across Asia showed the most frequently cited reason for women not using contraception was infrequent or no sex, with the prevalence of this reason substantially increasing in Nepal, Bangladesh and the Philippines, most likely due to increasing labour migration [62]. The 2016 NDHS stated approximately one-third of women in Nepal indicated their spouse lives away from the family home [8]. Multiple studies from Nepal suggest spousal separation, due to increasing male migrant workforce, is a prominent influencer of women’s uptake, continuation and/or discontinuation of contraception and could potentially play a role in Nepal’s stagnated contraceptive prevalence rate [51, 52, 54, 55, 63–65]. To ensure the contraceptive needs of Nepali women are met, family planning programs, SRH services and FCHVs must adjust their strategies to address the SRH needs of these couples [62].

Our findings document how women seek out safe or unsafe abortion services based on positive personal experiences or from the advice of the positive experiences of family and friends, with ease of access to services, concerns regarding confidentiality and economic burden also playing a role. Despite permissive laws and the government’s commitment to provide free safe abortion services, multiple Nepal based studies show awareness of the legal status of abortion and knowledge of safe abortion services remains low [8, 66–68]. Combined with lack of knowledge, barriers to access and uptake of safe abortion services will continue to facilitate demand for MA provision through pharmacies in Nepal [11]. It is essential SRH policymakers acknowledge the role pharmacies continue to play in the provision of MA and establish practical strategies to decrease negative health outcomes for women and increase access and referrals to safe MA [20, 61, 69].

The SRH and contraceptive information provided to women who seek MA through pharmacies is often inconsistent, inaccurate or non-existent with staff at times dispensing unsafe or ineffective forms of the drug [70, 71]. However, our study shows there is a proven desire for some pharmacy workers to provide support and quality of care for women seeking MA. Studies conducted in Nepal [20, 69] and other low- and middle-income countries [70, 72–77], highlight the challenges of implementing effective pharmacy based harm reduction strategies such as training and education of staff to help mitigate adverse health outcomes for women. Interventions to train pharmacy workers in harm reduction strategies in Zambia and Nepal demonstrated improvement in knowledge and referral practices [20, 69, 77], however, due to insufficient evidence of safety and effectiveness, current WHO guidelines do not recommend pharmacy staff provide MA [4].

Effective and safe MA provision by non-physician clinicians is well documented [56–60], with a recent Nepal based study demonstrating MA was as effective and safe when provided by trained auxiliary nurse-midwives at pharmacies as at government-certified health facilities [61]. As many mid-level health providers (nurses and auxiliary nurse-midwives) are proprietors of pharmacies in Nepal, their study details another promising avenue for safe and convenient MA provision and expansion through pharmacies [61].

While harm reduction strategies have the potential to increase accurate information provision and support relating to pharmacy supplied MA [20, 69], the sale of counterfeit brands or traditional medicines with purported abortive ingredients in the place of authentic MA will continue to contribute to the access of unsafe or ineffective medicine [20]. It is imperative that education regarding authentic Nepali registered brands, authentic but unregistered in Nepal brands, counterfeit MA brands and unsafe abortive medicines be a component of any pharmacy-based harm reduction training. Currently, there is limited global data on the types of MA pharmacies are providing within the WHO *less safe* and *least safe* framework [5, 78]. In Nepal, it is essential this be investigated within a broader, and much needed, comprehensive evidence base relating to MA provision through pharmacies to inform effective and functional policy.

Pharmacy-based harm reduction strategies have the potential to decrease morbidity and mortality relating to unsafe abortion as well as increasing access to MA, particularly in remote and rural settings that lack safe abortion services [11, 20, 69]. However, it is essential that effective contraceptive counselling including information provision, access to a range of contraceptive methods and established SRH service referral mechanisms for LARC insertion or permanent methods, be incorporated into these strategies to ensure women’s post-abortion contraceptive needs are met.

Global research has shown the effectiveness of MA self-management and remote support through telephone helplines, with approximately 20 safe abortion helplines currently active throughout Africa, Europe, Asia, and Latin America [71, 79–83]. With nearly all households in Nepal (93%) having access to at least one mobile telephone, remote support may provide another avenue of MA support for women and pharmacy workers [8]. Nepal based SRH helplines such as SPN/MSN’s *Meri Saathi* helpline can provide support to women and pharmacy workers with advice and information provided by trained SRH counsellors and clinical experts [84, 85]. As well as providing accurate SRH and MA information, trained and qualified counsellors can also ensure women receive comprehensive post-abortion contraception counselling along with referral to health facilities for contraceptive access and post-abortion complication support. Community-based health care workers such as FCHVs may also benefit from access to additional support through helpline services, ensuring their provision of accurate SRH and safe abortion information to women.

Our findings underscore the important role FCHVs play in the dissemination of SRH information within the community, with community-based health workers being the dominant community-based health *asset* cited throughout the cyclical AFRPAC framework of this study. Nepal based studies on FCHVs ability to assess MA eligibility and determine MA success concluded further refinement of tools are needed before effective and widespread use could be implemented [86, 87]. However, research shows that with effective SRHR training and ongoing education, community based health care workers like FCHVs can play a pivotal role in community-based SRH information provision, contraceptive counselling and access, and increasing awareness of safe abortion services and service referral [60, 88].

While every effort was made to mitigate bias in the research and to enhance credibility and trustworthiness, the study had some limitations. Although not by design, only married women who had already had at least one child at the time of MA access participated in interviews. Also, due to time constraints and access, CC participants were only sampled from one NGO SRH service (MSC) and did not include women who have accessed MA through government or private services. Recall bias due to the variable MA access timeline for PC respondents in comparison to CC participants is acknowledged.

## Conclusion

This qualitative research is an essential contribution to Nepal’s scarce evidence base on MA provision through pharmacies, providing a unique and in-depth analysis of the post-abortion experiences of women. The findings highlight the current disparity in the post-abortion care provision through safe abortion services and pharmacies in Nepal. Under current Nepali legislation, MA provision through pharmacies is considered unsafe and illegal, however through the implementation of innovative and effective harm reduction strategies, as well as access and information expansion strategies, the potential for increased access to safe MA throughout Nepal is evident. It is essential that post-abortion contraceptive counselling, access to a variety of contraceptive methods, and effective referral mechanisms to SRH services be a component of any strategies addressing MA provision through pharmacies.

## Additional files

Additional file 1: Thematic Analysis. (DOCX 18 kb)

## Data Availability

The qualitative data transcripts that support the findings of this study are not publicly available due to their content containing information that could compromise research participant privacy and consent. However, redacted versions may be made available from the corresponding author upon reasonable request.

## Abbreviations

AFRPA: Assets Focused Rapid Participatory Appraisal
CAC: Comprehensive Abortion Care
CC: Clinic Clients
FCHV: Female Community Health Volunteer
MA: Medical Abortion
MSC: Marie Stopes Centre
NHDS: Nepal Health and Demographic Survey
PAC: Post-abortion Care
PC: Pharmacy Clients
SPN/MSN: Sunaulo Parivar Nepal, implementing partner of Marie Stopes International Nepal
SRH: Sexual and Reproductive Health
SRHR: Sexual and Reproductive Health and Rights
