# Supplementary material for: Medical abortion in Nepal: a qualitative study on women’s experiences at safe abortion services and pharmacies
Source: Reprod Health. 2019 Jul 15;16:105. doi: 10.1186/s12978-019-0755-0 (PMC6632190; doi:10.1186/s12978-019-0755-0)
Supplement: Supplementary file 1 — Thematic Analysis. (DOCX 18 kb) [file 12978_2019_755_MOESM1_ESM.docx]

| **Additional File 1.**  ***Thematic Content Analysis of Interview Transcripts (n=20)*** | |
| --- | --- |
| **Overarching Themes**   1. Abortion Decision-Making Process 2. Medical Abortion Access and Uptake: Safe abortion services vs pharmacies 3. Medical Abortion Experience: Safe abortion services vs pharmacies 4. Post-Abortion Contraception and SRH Information Access and Uptake: Safe abortion services vs pharmacies 5. Contraception and SRH Information Access and Uptake: General | |
| **Themes** | **Sub-Themes** |
| **Abortion** |  |
|  |  |
| 1. Abortion Decision Making Process | 1. Sociocultural: Sex-Selection/Son Preference/2nd Trimester |
|  | 1. Sociocultural: Reproductive Coercion |
|  | 1. Socioeconomic: Poverty |
|  | 1. Socioeconomic: Education (of live children) |
|  | 1. Socioeconomic/Cultural: Empowerment |
|  | 1. Socioeconomic/Reproductive Health/Gender Roles: Child spacing |
|  | 1. Reason for Going to Clinic |
|  | 1. Reason for Going to Pharmacy |
|  | 1. MA Access Clinic: Participant Thoughts/Suggestions |
|  | 1. MA Access Pharmacy: Participant Thoughts/Suggestions |
|  |  |
| 1. Medical Abortion Experience | 1. Negative Abortion Experience: Pharmacy |
|  | 1. Negative Abortion Experience: Clinic |
|  | 1. Neutral Abortion Experience: Pharmacy |
|  | 1. Neutral Abortion Experience: Clinic |
|  | 1. Positive Abortion Experience: Pharmacy |
|  | 1. Positive Abortion Experience: Clinic |
|  | 1. MA Pharmacy: Complications Needing Follow-up at Clinic/Hospital |
|  |  |
| 1. Barriers to Safe Abortion Services (PC) | 1. Sociocultural: Stigma |
|  | 1. Socioeconomic: Poverty |
|  | 1. Geographic Isolation |
|  | 1. Governmental Policy |
|  | 1. Knowledge of Safe Abortion Services |
|  | 1. Unsafe Abortion: Participant Thoughts/Suggestions |
| **Contraception** |  |
|  |  |
| 1. Contraception Use (Past and Current) | 1. Negative Contraception Use Experience |
|  | 1. Neutral Contraceptive Use Experience |
|  | 1. Positive Contraception Use Experience |
|  | 1. Consistently Using Contraception |
|  | 1. Inconsistently Using Contraception |
|  |  |
| 1. Contraception Use at Conception | 1. Using Contraception When Fell Pregnant |
|  | 1. Not Consistently Using Contraception When Fell Pregnant |
|  | 1. Not Using Contraception When Fell Pregnant |
|  |  |
| 1. Contraception Information Access (MA use) | 1. Contraception Information: Clinic |
|  | 1. Contraception Information: Pharmacy |
|  | 1. No Contraception Information: Pharmacy |
|  |  |
| 1. Contraception Information Access (general) | 1. Professional: Medical (health post/clinic, SRH clinic, hospital) |
|  | 1. Professional: FCHV |
|  | 1. Non-Professional: Family, Friends, Neighbours |
|  | 1. Media |
|  |  |
| 1. Barriers to Contraception Access and Uptake | 1. Sociocultural: Reproductive Coercion |
|  | 1. Sociocultural: Gender Discrimination |
|  | 1. Socioeconomic: Poverty |
|  | 1. Geographic Isolation |
|  | 1. Governmental Policy |
|  | 1. Participant Thoughts/Suggestions |
|  |  |
| 1. Spousal Separation | 1. Husband Works in Local Community (lives in family home) |
|  | 1. Husband Works in Nepal (predominantly away from family home) |
|  | 1. Husband Works Internationally (predominantly away from family home) |
| **SRH Information** |  |
|  |  |
| 1. SRH Information Access (at time of MA) | 1. SRH Information: Clinic |
|  | 1. SRH Information: Pharmacy |
|  | 1. No SRH Information: Pharmacy |
|  |  |
| 1. SRH Information Access (general) | 1. Professional: Medical (health post/clinic, SRH clinic, hospital) |
|  | 1. Professional: FCHV |
|  | 1. Non-Professional: Family, Friends, Neighbours |
|  | 1. Media |
|  |  |
| 1. Barriers to SRH Information | 1. Sociocultural: Gender Discrimination |
|  | 1. Socioeconomic: Poverty |
|  | 1. Geographic Isolation |
|  | 1. Governmental Policy |
|  | 1. Participant Thoughts/Suggestions |
